# Books and Authors

**Published:** 1909-02

**Authors:** 


### BOOKS AND AUTHORS.

**General Surgery.** By **Ehrich Lexer,** M.D., Professor of Surgery in the University of Koenigsberg. American edition edited by **Arthur Dean Bevan,** M.D., Professor and head of the Department of Surgery, Rush Medical College, (University of Chicago). An authorised translation of the second German edition by **Dean Lewis,** M.D., Assistant Professor of Surgery, Rush Medical College. Octavo, pp. 1041. With 449 illustrations and two colored plates. New York, and London: D. Appleton & Co.

Three elements conspire to make this treatise of value,—author editor, and translator. The first is a German, hence it may be regarded as an exposition of the status of surgery in Germany. It must be remembered, however, that the editor is one of the leading surgeons in America, hence its adaptation to conditions existing in this country are as complete as may well be made. The translator has made smooth English of the German text.

This treatise, as announced by the editor, is a presentation of the scientific principles upon which the practice of modern surgery is based. Bevan maintains that the subject of general surgery should be studied before the student takes up regional or special surgery, a dictum with which we quite agree. It is on this foundation that the teachings of this book rests.

The work covers a vast field which once mastered equips the student with a knowledge of the fundamentals of surgery, including injuries and their repair, new growths, wound infection, aseptic technic, and everything of importance or value before he takes up regional or special surgery.

It is by far the most striking surgical treatise that has been issued in years; indeed, never before in this country has such an admirable exposition of surgical science been put forth. The editor and all his associates, including the publishers, deserve the thanks of the profession in general for the admirable work he has done in preparing this volume for the American students and practitioners of surgery.

---

**Progressive Medicine,** Vol. X., No. 4, December 1908. A Quarterly Digest of Advances, Discoveries and Improvements in the Medical and Surgical Sciences. Edited by **Hobart Amory Hare,** M.D., Professor of Therapeutics and Materia Medica in the Jefferson Medical College of Philadelphia, assisted by **H. R. M. Landis,** M.D. Octavo, 333 pages, with 28 engravings. Lea & Febiger, Publishers, Philadelphia and New York. (Per annum, in four cloth-bound volumes, $9.00; in paper binding, $6 00, carriage paid to any address.)

This number deals with diseases of the digestive tract and allied organs, the liver and pancreas. by David L. Edsall; diseases of the kidneys, by John Rose Bradford; surgery of the extremities, tumors, surgery of joints, shock, anesthesia, and infections, by Joseph C. Bloodgood; and genitourinary diseases, by William T. Belfield. A practical therapeutic referendum, by H.

R. M. Landis is added. The digests are well prepared and give essential information on the topics presented. We were greatly interested in the therapeutic referendum by Landis. It contains a vast amount of useful information concerning various drugs and remedies, the serums, the nitrites, and various other treatment aids. The tuberculosis problem receives attention, and in many respects this is one of the better issues of this periodical. It is a most useful book for the general practitioner.

---

**Anatomy, Descriptive and Surgical.** By **Henry Gray,** F.R.S., late lecturer on Anatomy at St. George's Hospital, London. New American edition, enlarged and thoroughly revised, by **J. Chalmers Da Costa,** M.D., Professor of Surgery and Clinical Surgery, and **Edward Anthony Spitzka,** M.D., Professor of Anatomy, in the Jefferson Medical College of Philadelphia. Imperial octavo, 1,625 pages, with 1,149 large and elaborate engravings. Lea & Febiger, Publishers, Philadelphia and New York, 1908. (Cloth, $6.00; leather, $7.00, net prices.)

Anatomy, formerly considered a "dry" study, has become under the stimulus of more recent authors and teachers, even fascinating. These men have infused the topic with new interest by their methods and works. Gray led the advance in creating a treatise that was both novel and interestingly instructive, and he held the pace during his lifetime. Now his successors, so to speak, Da Costa and Spitzka, have continued the work keeping it well in the lead, as an examination of this edition will show.

One might easily presume the changes since the last edition of this work would be few, but in this edition there have been many alterations, eliminations, and some additions of important anatomical facts, besides changes in and improvements of the illustrations. Gray, always a favorite with students, has been never more so than now. The illustrations are superb, many being in colors, and all sufficiently accurate in detail to depict the parts or structures to the understanding of the pupil. The text, too, is clear and simple, enabling the student to create a mental picture of the structures or tissues described. Taken as a whole, Gray is still one of the best anatomical books for the practical physician.

---

**International Clinics.** A Quarterly of Illustrated Clinical Lectures and especially prepared articles on Treatment, Medicine, Surgery, Neurology, Pediatrics, Obstetrics, Gynecology, Orthopedics, Pathology, Dermatology, Ophthalmology, Otology, Rhinology, Laryngology, Hygiene and other topics of interest to students and practitioners. Edited by **W. T. Longcope,** M.D., Volume III, eighteenth series. Philadelphia and London: J. B. Lippincott Co. 1908. (Cloth, $2.00.)

The subjects considered in this number are treatment, three articles; medicine, four articles; surgery, four articles; gynecology, one article; pediatrics, two articles; orthopedics, two articles; psychiatry, two articles; neurology, two articles; ophthalmology, three articles; rhinology, one article; and pathology, one article. The most noted contributor is Sir Dyce Duckworth (London), whose lecture on sciatica, however, is short considering the importance of the subject. The most notable article, probably, is that of Jastrow (Univ. Wisconsin), entitled, "On the trail of the subconscious."

The article on chronic milk infection by Richard B. Gilbert, and one on adenoid vegetations in the nasopharynx, by J. Morrison Ray, are worthy of mention. Taken together the book contains much valuable material though its illustrations are not quite up to the usual standard.

---

**Modern Medicine.** Its Theory and Practice. In original contributions by American and Foreign authors. Edited by **William Osler,** M.D., Regius Professor of Medicine in Oxford University, England; formerly Professor of Medicine in Johns Hopkins University, Baltimore; in the University of Pennsylvania, Philadelphia, and in McGill University, Montreal. Assisted by **Thomas McCrea,** M.D., Associate Professor of Medicine and Clinical Therapeutics in Johns Hopkins University, Baltimore. In seven octavo volumes of about 900 pages each, illustrated. Volume V., Diseases of the Alimentary Tract. Price per volume: cloth, $6.00, net; leather, $7.00, net; half morocco, $7.50, net. Lea & Febiger, Publishers, Philadelphia and New York, 1908.

This book makes the fifth of the seven volumes promised by the publishers. It deals with diseases of the alimentary tract, and is composed of ten chapters, each prepared by a different author. The first chapter, being an introductory discussion on the diseases of the digestive apparatus, is written by Charles G. Stockton, of Buffalo, than whom none is more competent. Indeed, we could wish that Professor Stockton had not limited his writing to this chapter, but had dealt with diseases of the stomach, functional and organic, and of the intestines. However, this might have interfered with the general scheme of the editor.

The other chapters are written by Reisman, Philadelphia, whose subject is diseases of the mouth and salivary glands; McCrae, Montreal, who presents diseases of the esophagus; Friedenwald, Baltimore, his topic being functional diseases of the stomach; Martin, Montreal, who deals with organic diseases of the stomach; Stengel, Philadelphia, who handles diseases of the intestines; Rolleston, London, who writes of diseases of the peritoneum; Brown, Baltimore, whose topics are splanchnoptosis, enteroptosis, and Glenard's disease; Opie, Baltimore, who discusses diseases of the pancreas; and Kelly, Philadelphia, who considers diseases of the liver, gall-bladder, and biliary ducts.

These several sections are carefully prepared, presenting the topics clearly, and after the most modern thought on the various diseases, relating to cause, pathology, symptoms, diagnosis, course, prognosis, and treatment. It is a fitting companion to the previous volumes in the series.

**Pathogenic Microörganisms, including Bacteria and Protozoa.** A practical manual for students, physicians and health officers. By **William H. Park,** M.D., Professor of Bacteriology and Hygiene in the University and Bellevue Hospital Medical College, New York. New (third) edition, thoroughly revised and much enlarged. Octavo, 648 pages, with 176 illustrations and 5 full-page plates. (Cloth, $3.75, net. Lea & Febiger, Philadelphia and New York, 1908.

The author of this work has contributed much to the knowledge of bacteria and protozoa. As director of the research laboratory of the department of health in New York he has studied much, learned much, and imparted much. Whoever is placed at the head of such a laboratory must be competent, and he must work. Park has demonstrated his capacity in this line of research work and, besides, was almost the first to put his work into book form for the benefit of others.

This new edition, the first since three years ago, represents the growth of the subjects during that period, which has been considerable. It has necessitated revision and enlargement, so the book now is increased in size. Subjects heretofore thought but little of, such as the opsonic index, and the bacteriology of the normal intestine are now taken up and considered at length. It is a book for both physician and student in their studies of pathogenic microörganisms, and one of the better of its kind.

---

**Surgical Memoirs and Other Essays.** By **James G. Mumford,** M.D., Instructor in Surgery, Harvard Medical School. 12 mo, pp. 358. Illustrated. New York: Moffat, Yard & Co. (Cloth, $2.50.)

These memoirs embrace a collection of essays, addresses, and classic narratives. They give sketches of Hippocrates, Galen, Vesalius, Ambrose Paré, von Haller, John Hunter and Joseph Lister. Then, under the title "American Surgery," are short biographies of the earlier surgeons in this country. A section deals with the teachings of the older surgeons. An admirable estimate of the life and character of Sir Astley Cooper is next given, which should be read by every junior in the ranks of medicine. Then follow sketches of Sir Benjamin Brodie, John Collins Warren, and Jacob Bigelow.

The remainder of the volume is taken up with Boston medicine one hundred years ago; studies in aneurism; present problems; an address to nurses; the nurses' vocation; and the history and ethics of medicine. Though the grouping is somewhat incongruous, the book is an interesting one and will appeal to every lover of literature pertaining to the fathers of medicine, as well as of medical men of more recent periods.

---

**General Pathology.** By **Dr. Ernst Ziegler,** Breisgau. Eleventh revised German edition. Edited by **Aldred Scott Warthin,** M.D., Ann Arbor. Octavo, pp. 801. Illustrated. New York: William Wood & Co. (Cloth, $5.50.)

Three years ago the eleventh edition of this work left the author's hands, during which time pathological knowledge has advanced in a marked degree. The editor, therefore, in order to bring the work forward to embrace those facts that have proven of value, has added material in fine print to the paragraphs or chapters where needed. These interlardments include recent observations on the effects of *x*-rays and on heredity, phagocytosis, opsonins, blood-plates, thrombosis, necrosis, cloudy swelling, fatty degeneration, calcification, regeneration, inflammation, malignant neoplasms, tuberculosis, syphilis, relapsing fever, spirochetæ, protozoa, and the like. Zieglers' pathology has been regarded as a classic so long that it is scarcely possible for the well-informed student to do without it, and with these additions by the American editor it becomes more indispensable than ever.

---

**Gynecology and Abdominal Surgery.** In two volumes. Edited by **Howard A. Kelly,** M.D., Professor of Gynecologic Surgery at Johns Hopkins University; and **Charles P. Noble,** M.D., Clinical Professor of Gynecology at the Woman's Medical College, Philadelphia. Large octavo volume of 862 pages, with 475 original illustrations by **Mr. Hermann Becker** and **Mr. Max Brodel.** Philadelphia and London: W. B. Saunders Company, 1908. (Per volume: Cloth, $8.00; half morocco, $9.50 net prices.)

The second volume of this colossal work is even more absorbing than the first, which is saying much in its praise. The chapter by Ross (Toronto), dealing with Cesarean and Porro Cesarean section is of first importance. It deals with conditions likely to be met in the lying-in room at any time and it tells of the conditions justifying or rendering imperative these procedures, or operations. With the perfected technic of the present day and operation before the exhaustion of the mother, the maternal mortality is very much reduced and Ross, himself, has contributed not a little to make the reduction in the mortality.

The chapter on extrauterine pregnancy by Williams (Baltimore), is an exposition of this subject from the days when Lawson Tait (1883) first operated for tubal pregnancy, to the present time, complete in detail and instructive in method. Tait's "Lectures on Ectopic Pregnancy and Pelvic Hemotocele," (1888) furnish the foundation for an intelligent bibliography relating to this anomaly of nature, around which has centered whatever knowledge we now possess concerning it.

Only less important, even scarcely so, is the chapter on diseases of the breast, by Bloodgood (Baltimore), in which pathology is given prominence as it should be. All affections of the breast from mastitis to sarcoma are described, treated, and operated in excellent form. Murphy's (Chicago) chapter on intestinal surgery is, like all of his work, splendidly done and profusely as well as graphically illustrated. Kelly, himself, in association with Elizabeth Hurdon, writes of appendicitis and of operations pertaining to its cure. It is needless to add that this chapter is beautifully as well as accurately illustrated.

The chapter on tuberculosis of the peritoneum by Johnston (Richmond, Va.), sets forth the history, clinical picture, and operative treatment of this important malady. He credits Van de Warker (Syracuse), as first inviting attention in this country to the operative treatment of the peritoneum. His paper was published in the *American Journal of Obstetrics,* 1887 The chapter is most interesting and instructive. The chapters on the surgery of the pancreas (Opie-Watts), on operations during pregnancy, (Norris), surgery of the kidney (Noble-Anspach) and several others of which we would like to make mention, but which, perforce, we are compelled to omit by reason of scant time, are all worthy the highest praise. The book, as a whole. is a valuable contribution to gynecology and abdominal surgery, and will take its place as such in the forefront of literature on those topics.

---

**Operative Midwifery,** by **J. M. Munro Kerr,** M.B., C.M., Glas. Fellow of the faculty of physicians and surgeons, Glasgow; obstetric physician to Glasgow Maternity Hospital; gynecologist to the Western Infirmary, etc., with 294 illustrations in the text. New York: William Wood & Company. 1908. (Price, $6.50 net. Cloth.)

Operative obstetrics is about the only kind of obstetrics men in middle life care to practise. Barnes, in his lectures on obstetric operations, led the way in this kind of literature, and his work may be regarded as a classic. Herman's monograph on difficult labor, coming a few years later, supplied an important link in the chain. But from Barnes to Kerr is a long stride, so many things have happened since the last edition of the former (1886).

Formerly operations on the pregnant or parturient woman were made only under most direful conditions; whereas, nowadays it is almost criminal to wait for extreme indications before surgical intervention is taken. A study of Kerr will engender that courage which always needs support in emergencies. He gives the approved technic of the operations that may be necessary and tells how the diagnosis may be made. The book, therefore, becomes quite as useful to the obstetrician, pure and simple, as to the obstetric surgeon. The former must be able to tell when the operation is needed, even should he not be able to make it himself; he must know that he may send for the surgeon at the opportune moment, before the patient,—the patients,—are beyond surgical relief.

The engravings are graphic, depicting the procedures in most excellent form. The book is a distinct addition to permanent medical literature, and especially to the surgery of midwifery.

---

**High-Frequency Currents** by **Frederick Finch Strong,** M.D. Instructor in Electro-therapeutics at Tuft's College Medical School, Boston. With 183 illutrations in the text. Rebman Company, 1123 Boardway, New York.

A reading of this book will benefit every one who does not understand high frequency currents. It contains much of value relating to electrons, vibration, electrophysics, and various collateral scientific factors pertaining to use of these electric currents. There is nothing new or reliable, however, as to the therapeutic application of this form of electricity. The author starts off with a preface rather high pitched and the entire book overflows with italics and an unnecessary use of capital letters. The printer must have been perplexed with the "copy" and glad when done with it. The author, however, promises a sequel to this book in two years, and then more trouble for the printer.

---

**A Textbook of Physiology.** For Students and Practitioners. By **George V. N. Dearborn,** A.M., (Harvard), Ph.D., M.D. (Columbia), Professor of Physiology in Tufts College, Medical and Dental Schools, Boston. Octavo, 550 pages, with 300 engravings and 8 colored plates. Lea & Febiger, Publishers, Philadelphia and New York, 1908. (Cloth, $3.75 net.)

Physiology next to anatomy lays the foundation of all medical knowledge, all medical skill. This new work by a new author presents the subject in a new form. Dearborn emphasises the mechanism of the sense organs, nerves, and muscles, as the basis of the individual's efficiency. It is believed to be the first textbook of medical physiology to recognise the ever growing insistent demands of the mental process. For these and other reasons the work is especially adapted to the requirements of students as well as teachers of physical education and of psychology. Dearborn presents the topic in an attractive manner, without undue verbosity and is certain to win his way to the very front line of teachers of physiology.

---

**Pathological Technic.** By **Frank Burr Mallory,** M.D., and **James Homer Wright,** M.D., Assistant Professors of Pathology, Harvard University Medical School. Fourth edition. Octavo, pp. 480. Illustrated. Philadelphia and London: W. B. Saunders Co. (Cloth, $3.00.)

This work has been in the hands of the profession, at least that portion interested in pathologic laboratory work, since 1897, during which time four editions have been issued. It was designed as a practical guide to the student in the laboratory and as a reference book for those more advanced; it has kept to these lines in its later editions. It has been revised, enlarged, and improved until now it is about the best known and most valued book of its kind extant. This edition has been revised to meet changes in methods and, likewise, has sustained such additions as are needed to keep it in the advance line of pathologic investigation. The directions for the examination of the dead body are complete and should be followed by post mortem examiners. It is a work that should be found in the hands of every student and teacher of pathology.

**The Principles of Pathology.** Volume I., General Pathology. By **J. George Adami,** M.A., M.D., LL.D., F.R.S., Professor of Pathology in McGill University, Montreal. Octavo, 948 pages with 322 engravings and 16 plates. Lea & Febiger, Publishers. Philadelphia and New York, 1908. (Cloth, $6.00 net.)

It is quite impossible to do adequate justice to this colossal work in the scant space at command in this magazine, with so many books clamoring, so to speak, to be reviewed,—at all events waiting review. It is not difficult, however, to review the author, which is of equal importance, perhaps even of greater moment.

Professor Adami has long been recognised as one of the foremost pathologists of the day, and perhaps it is not too much to accord him first place on the North American continent. Certainly he is the representative Anglo-Saxon pathologist of the present period. It is twelve years since this work was projected and during that time the author has met and overcome obstacles that would have dismayed a less capable man,—a man with less energy and less courage. But Adami had something to say and he has said it in a forceful manner,—in a fashion that will make it a lasting monument to his name.

It is a stupendous undertaking to prepare and bring out a work on general pathology like this one; nevertheless, Adami has succeeded in putting forth what may be regarded, judging by the enormous value of the first volume, as the best English language work on pathology yet printed. It is vastly more difficult to interpret correctly the phenomena of disease, which is the province of pathology, that the phenomena of health which belongs to the field of physiology, to illustrate the difficulties Adami has met and overcome in the preparation of his books, his manuscript had been prepared and all but a few chapters sent to the publishers when, in April, 1907, the medical building at McGill University was burned. The chapters referred to, his library, and the illustrations made or collected for this book were destroyed. One can readily imagine what a blow this was to the author. But in spite of all this, he has produced a wonderful work and the illustrations are well chosen, mostly from other sources.

This author has proved himself a conscientious follower of Virchow in his studies of cell life and cell growth. This is wise, as no better guide in this regard than the great German pathologist has appeared up to the present time. The whole work, indeed, is rather an exposition of ascertained facts, than a propagandist of novel theories.

We could wish that every teacher of pathology would adopt Adami's Treatise as a guide and that advanced students would be sure to make use of it in their further investigations. It is safe and scientific, and redounds to the credit of English speaking investigators. If we mistake not it will be adopted in all the schools of medicine on this side the water as a textbook.

**Spectacles and Eyeglasses,** their forms, mounting, and proper adjustment, by **R. J. Phillips,** M.D., ophthalmologist to Presbyterian Orphanage, etc. Fourth edition, revised, with 56 illustrations. Philadelphia: P. Blakiston's Son & Company, 1012 Walnut street. 1908. (Price, $1.00 net.)

A monograph of this kind is useful to the beginner in refraction as it is of convenient form to make of easy reference. Moreover, it is an inexpensive book and new editions, frequently appearing, keep the refractionist promptly advised of the improvements constantly making not only in the methods of proper adjustment of spectacles and eyeglasses, but of the advance in mechanical optics. In this edition we particularly note the description of "invisible" bifocals, and other devices of both benefit and convenience to the wearer of lenses. Phillips is an expert at this work and his book is both practical and scientific.

